# Use of Lipid-Lowering Treatment in Primary Prevention in Spain (Lipidspain)

**DOI:** 10.3390/jcm14176059

**Published:** 2025-08-27

**Authors:** Miguel García-Villarino, Claudia Lozano-Aida, Lorena Suárez-Gutiérrez, Carmen Lambert, Tomás González-Vidal, Ana Victoria García, Pedro Pujante, Elsa Villa-Fernández, Elías Delgado-Álvarez, Edelmiro Menéndez-Torre, Jessica Ares-Blanco

**Affiliations:** 1 Instituto de Investigación Sanitaria del Principado de Asturias (ISPA), Edificio ISPA-FINBA, Planta N-1, F-1.64 Avda. del Hospital Universitario, s/n, 33011 Oviedo, Spain; garciavmiguel@uniovi.es (M.G.-V.); pedropujanteal@gmail.com (P.P.); eliasdelga@gmail.com (E.D.-Á.); edelangot@gmail.com (E.M.-T.); jessiaresb@gmail.com (J.A.-B.); 2 Department of Medicine, University of Oviedo, 33003 Oviedo, Spain; 3 Instituto Universitario de Oncología del Principado de Asturias (IUOPA), 33006 Oviedo, Spain; 4 Servicio de Endocrinología y Nutrición, Hospital Universitario Central de Asturias (HUCA), 33011 Oviedo, Spain

**Keywords:** lipid-lowering drugs, BDCAP, lipids, primary prevention

## Abstract

**Background/Objectives**: Cardiovascular disease remains a leading cause of mortality in Spain, with dyslipidemia being a major modifiable risk factor. Lipid-lowering therapy (LLT) is essential for cardiovascular risk reduction, but regional disparities in prescription patterns and LDL-C control persist. This study analyzes LLT prescription trends in Spain in 2019 and 2023, assessing temporal, demographic and regional differences. **Methods**: A retrospective observational study was conducted using the Spanish Primary Care Clinical Database (BDCAP), which contains 4.8 million anonymized primary care records. LLT prescriptions for primary prevention were analyzed by sex, age, community size, and employment status. Trends from 2019 to 2023 were evaluated, distinguishing between monotherapy and combination therapy. **Results**: In 2023, 5.8 million individuals received LLT for primary prevention (139.6 per 1000). Women had higher treatment rates than men after age 60. Treatment rates were highest in small communities and among retirees. The use of combination therapies almost doubled from 2019 to 2023, achieving better LDL-C control (56.4% vs. 41.5% with monotherapy, *p* < 0.001). Regional disparities were evident, with the lowest treatment rates in Cataluña and País Vasco and the highest in Galicia. **Conclusion**: LLT prescription patterns in Spain show remarkable socioeconomic and regional disparities. The increase in combination therapy suggests a shift towards more intensive lipid management. Standardized guidelines and targeted interventions are needed to ensure equitable and effective dyslipidemia treatment.

## 1. Introduction

Cardiovascular diseases, which have historically led to the highest number of deaths in Spain, have recently been surpassed by tumors as the main cause of mortality. According to provisional data from the National Institute of Statistics (INE) for 2023, the number of deaths from cardiovascular diseases in Spain is expected to increase in 2023 [1]. Over the past decade, tumors have accounted for 26.6% of deaths, while diseases of the circulatory system have accounted for 26.5%. This shift is indicative of substantial progress in the prevention and management of cardiovascular pathologies, including more effective control of risk factors such as dyslipidemia. Despite this transition, it is crucial to maintain control of dyslipidemia since hypercholesterolemia is directly associated with the development of cardiovascular events. In the context of primary prevention, aimed at patients without established cardiovascular disease, lipid-lowering therapy plays a crucial role in reducing low-density lipoprotein cholesterol (LDL-C) and, thus, cardiovascular risk [2,3].

In Spain, 50% of the adult population has total cholesterol levels above 200 mg/dL [3]. However, LDL-C control rates vary between Autonomous Communities (AC), due to the decentralization of healthcare competencies. This has led to disparities in access to treatment, the implementation of clinical guidelines, and the achievement of therapeutic targets [1,2]. These differences highlight the necessity for specific strategies to ensure equitable care throughout the national territory. Inequities in access to and utilization of healthcare systems, along with the characteristics of population health status, position place of residence as one of the most significant determinants of health [4,5]. In the context of the Spanish Healthcare System (SHS), where competencies in health are devolved to the Autonomous Communities, strategies aimed at cardiovascular prevention exhibit marked heterogeneity.

In this regard, a national study analyzing the care pathways for patients with dyslipidemia identified geographic differences as the main difference, while also highlighting limited coordination in the implementation of shared protocols across different levels of care [6]. However, uncertainty remains regarding the existence of regional disparities in cholesterol control, particularly among patients at high or very high cardiovascular risk.

Despite advances in the management of dyslipidemia, knowledge gaps persist regarding the impact of these regional differences on the primary prevention population, particularly in relation to sex, age, or geographic region. This study aims to analyze the prescription of lipid-lowering agents in Spain in 2019 and 2023, assessing temporal, demographic and regional differences and identifying patterns that may inform more effective strategies for cardiovascular risk prevention.

## 2. Material and Methods

### 2.1. Contextualization, Data Request and Eligibility Criteria

The Primary Care Clinical Database (BDCAP) refers to a systematically and homogeneously collected dataset that provides a longitudinal perspective on clinical care delivered at the primary care level. This information is sourced directly from electronic health records (EHRs), specifically the EHRs used in Primary Care settings, and includes clinical data from a random sample of 4.8 million primary care health records. The most recent update of the database, in 2023, encompasses data from individuals assigned to Primary Care services of the SHS across the entire country. This analysis incorporated demographic and socioeconomic variables, along with LDL-C levels, to provide a detailed characterization of the population receiving lipid-lowering therapies in primary prevention. These are anonymized and coded using standardized systems, such as the International Classification of Diseases (ICD9, ICD10ES) and the International Classification of Primary Care (CIAP2) for health conditions, and the National Code for drugs. Sampling was performed using single-stage random sampling by clusters (basic health areas), stratified by autonomous community and the municipality where health centers are located. The BDCAP is representative of the population holding an individual healthcare card (TSI) and aims to provide insights into what is addressed in Primary Care, how it is managed, the outcomes achieved, and the associated costs. We conducted a retrospective observational study without interventions, utilizing the statistical portal provided by the Ministry of Health, which is associated with the BDCAP database [7]. Although the BDCAP is based on a representative sample of approximately 4.8 million EHRs from Primary Care, the data analyzed in this study were obtained through the Ministry of Health’s statistical portal, which extrapolates the results to the entire population assigned to the SHS. This allows for nationally representative estimates stratified by Autonomous Community, age, sex, and other demographic characteristics. According to the extrapolated data for the year 2023, the total assigned population was 46.7 million individuals. Of these, 7.3 million received at least one lipid-lowering prescription, and 5.8 million of them were classified as being treated in the context of primary prevention [7].

### 2.2. Study Variables

The BDCAP database is structured into separate information cubes, each corresponding to a specific analytical or study variable. Currently, seven variables are available for analysis: health problems, comorbidities, consultations, drugs, visits, procedures, and parameters. For the present study, we used the “drugs” entry of the statistical portal associated with the BDCAP database. This entry includes comprehensive records of medications that were actively prescribed and/or dispensed at the time of the study, as documented in patients’ electronic medical records. All medications are coded using the standardized National Code, ensuring consistency and comparability across the dataset. For the purpose of this study, individuals were classified as receiving lipid-lowering therapy for primary prevention if they had no recorded diagnosis of major cardiovascular disease during the study year. Cardiovascular disease was defined based on the presence of coded diagnoses such as myocardial infarction, stroke, peripheral arterial disease, or other atherosclerotic events, as recorded in the electronic health record.

Our analysis focused specifically on lipid-lowering agents categorized under the Anatomical Therapeutic Chemical (ATC) classification code C10A. However, the BDCAP statistical portal does not disaggregate the data by specific pharmacological subgroups within this code, such as statin molecule, intensity (low, moderate, high), or co-administration with ezetimibe, PCSK9 inhibitors, or other agents. Therefore, our analysis reflects overall prescription patterns of lipid-lowering drugs rather than specific drug classes or regimens. Information on drug dispensing or pharmacy claims was not available in the dataset; therefore, results refer to prescribed treatments and not to confirmed dispensing or patient acquisition. We then analyzed prescribing patterns across the population to gain insights into the use of these therapies. The study focused on the prevalence of lipid-lowering drug prescriptions stratified by gender (male and female) and age. To facilitate age-based analyses, the predefined age categories provided by the BDCAP database were employed, which categorized individuals into the following age groups: 30–34 years, 35–39 years, 40–44 years, 45–49 years, 50–54 years, 55–59 years, 60–64 years, 65–69 years, 70–74 years, 75–79 years, 80–84 years, 85–89 years, 90–94 years, and ≥95 years.

Additionally, sociodemographic factors were examined to identify disparities in treatment patterns. These included the size of the municipality based on population (<10,000, 10,001–50,000, 50,001–100,000, 100,001–500,000, and >500,000 inhabitants), and employment status, which was categorized as follows: employed, unemployed, inactive, retired, or other. These variables allowed for a thorough evaluation that provided a comprehensive assessment of treatment variability across different socioeconomic groups.

The analysis also included treatment type, distinguishing between single-drug and combination therapies. This differentiation enabled the evaluation of trends in the utilization of combination regimens, which have become increasingly relevant for achieving stricter lipid control. Temporal trends were assessed over a five-year period from 2019 to 2023, with an emphasis on the evolution of treatment prevalence and defined daily doses (DDD) per 1000 individuals per day. LDL-C outcomes were evaluated to compare the efficacy of single-drug versus combination therapies in achieving lipid control. The proportion of patients reaching LDL-C levels below 100 mg/dL was calculated and analyzed to assess treatment effectiveness. This analysis incorporated demographic and socioeconomic variables, along with LDL-C levels, to provide a detailed characterization of the population receiving lipid-lowering therapies in primary prevention.

### 2.3. Statistical Analyses

Quantitative variables are expressed as absolute values (e.g., the number of individuals with health problems or taking lipid-lowering drugs) and as percentages relative to the total population or the specific reference subpopulations. For age-stratified and gender-specific analyses, results are presented as the number of cases per 1000 individuals assigned to the SNS. This approach provides a standardized measure to compare prevalence rates across different demographic and regional categories. Group comparisons were performed for categorical variables (e.g., gender, employment status) using chi-square tests, while continuous variables were analyzed using *t*-tests or their non-parametric equivalents (Mann–Whitney U test) depending on data distribution. For all analyses, statistical significance was set at *p* < 0.05.

Temporal trends in the use of lipid-lowering therapies (single-drug vs. combination) and DDD per 1000 individuals per day were analyzed between 2019 and 2023. Subgroup analyses were conducted to explore differences in LDL-C outcomes, particularly the proportion of patients achieving LDL levels below 100 mg/dL, stratified by treatment type (single vs. combination therapy). Additionally, regional analyses evaluated discrepancies in treatment prevalence and combination therapy use across Spain’s Autonomous Communities.

## 3. Results

In 2023, a total of 7,373,297 people were treated with lipid-lowering drugs, representing 157.7 per 1000 people assigned to the SHS in Spain. This figure was 166.6 per thousand in women and 156.6 per thousand in men. Most of these patients (140.5 per thousand people assigned) are taking single drugs while 24.2 per thousand people are taking drugs in combination (Table 1). A total of 5,807,868 individuals were treated for primary prevention, with an attendance rate of 139.6 per thousand. Furthermore, 1,565,429 individuals were treated for secondary prevention, with an attendance rate of 678 per thousand (Table 1). The findings indicated substantial variation in the use of lipid-lowering medications based on population size and employment status (Table 2). When analyzed by municipality size, individuals living in smaller municipalities with <10,000 inhabitants had the highest treatment rates (162.4 per 1000), while the lowest rates were observed in larger cities with >500,000 inhabitants (119.7 per 1000; *p* < 0.001). Regarding employment status, retired individuals showed the highest treatment rate (400.5 per 1000), followed by unemployed individuals (121.9 per 1000). In contrast, the lowest treatment rates were seen among inactive individuals (51.6 per 1000) and employed individuals (82.9 per 1000) (*p* < 0.001). A temporal analysis from 2019 to 2023 (Table 3) revealed a gradual increase in the number of individuals receiving lipid-lowering treatment. This trend was accompanied by an increase in the DDD, indicating intensified therapeutic strategies. Notably, the use of combination therapies showed a pronounced rise, increasing from 7.25 per 1000 individuals assigned in 2019 to 15.5 per 1000 in 2023, reflecting a shift toward combination treatments to improve lipid control.

**Figure 1:**
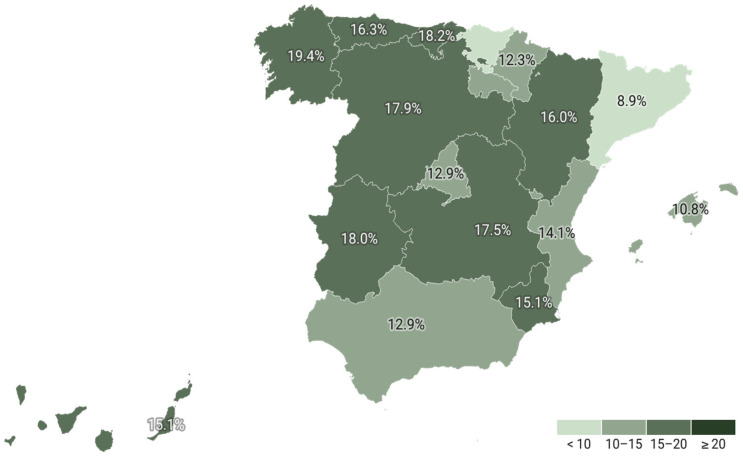
Percentage of persons with lipid lowering drugs by region in primary prevention.

**Figure 2:**
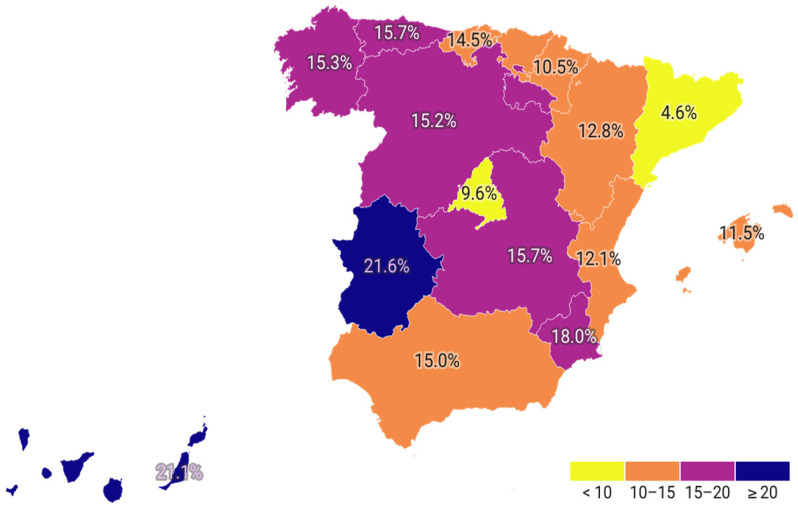
Percentage of the population treated with lipid-lowering therapy receiving combination treatment, by region in primary prevention.

As shown in Table 4, the majority of patients treated for primary prevention were over 60 years of age, with a steady increase in treatment prevalence until 80 years of age, after which it began to decline. The data indicates that the percentage of men receiving treatment was higher than that of women up to the age of 60 years. However, after the age of 60, the trend reverses, with a higher percentage of women receiving treatment. Regional analyses revealed notable discrepancies in the use of lipid-lowering drugs across Spain’s Autonomous Communities (Table 5 and Figure 1). The lowest treatment rates were observed in Cataluña (8.9%) and País Vasco, while the highest rates were seen in Galicia (19.4%). The use of combination therapies also varied widely between regions, ranging from 7.97% in Cataluña to 20.23% in Canarias (Figure 2).

**Figure 3:**
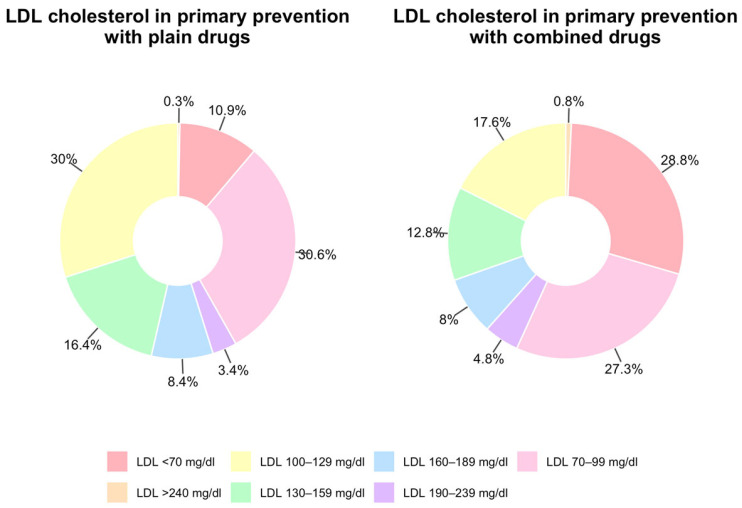
Intervals of LDL-cholesterol achieved and type of lipid-lowering treatment in primary prevention.

Regarding plasma LDL cholesterol levels among patients treated for primary prevention, the results demonstrated that combination therapies achieved superior lipid control. Specifically, 56.4% of patients receiving combination treatments achieved LDL levels below 100 mg/dL, compared to 41.5% of those receiving monotherapy (Figure 3).

## 4. Discussion

This study on the use of lipid-lowering therapies in primary prevention in Spain provides valuable insights into the current state of dyslipidemia management, as defined by the current ESC/EAS clinical guidelines for cardiovascular risk assessment and lipid control strategies [8]. There has been a notable increase in the prescription of these treatments, with trends varying by sex, age, and socioeconomic context. Notably, women had higher treatment rates than men, particularly after the age of 60. Socioeconomic disparities were evident, with higher treatment rates observed among individuals living in smaller municipalities. The increasing use of combination therapies indicates a shift toward more intensive lipid management strategies, which has been shown to achieve better LDL-C control compared to monotherapy, but significant regional disparities remain, highlighting the need for targeted interventions to ensure equitable access to lipid-lowering therapies throughout Spain.

The substantial number of individuals receiving lipid-lowering therapies for primary prevention, with over 5.8 million people treated in 2023, corresponds to an attendance rate of 139.6 per 1000 individuals registered in the SHS. The upward trend in both the number of individuals receiving these treatments and the DDD from 2019 to 2023 underscores a growing emphasis on lipid control as a cornerstone of cardiovascular risk management. The higher treatment rates observed in men compared to women, particularly in younger age groups, suggest gender-specific differences in prescribing patterns. These findings are consistent with prior research indicating that women are less likely than men to be prescribed statin therapy. Additionally, when prescribed, women often receive lower-intensity statins, which can contribute to suboptimal lipid control in younger women [9]. In addition, research has shown that women are more likely to discontinue statin therapy due to adverse effects, which could potentially exacerbate existing disparities in lipid management between the sexes [10]. However, this trend reverses after the age of 60, with higher treatment rates observed in women. This phenomenon could be attributed to the increased cardiovascular risk associated with menopause. Postmenopausal women experience unfavorable changes in lipid metabolism, including increased LDL-C and total cholesterol levels and decreased HDL-C levels, which contribute to a higher atherogenic profile and cardiovascular risk compared to premenopausal women [11,12]. These hormonal and metabolic changes may necessitate more proactive lipid-lowering interventions in this population, particularly in the context of primary prevention [13].

Differences in prescription rates of lipid-lowering therapies were observed across geographic areas, with higher rates observed among those residing in smaller municipalities, reflecting regional and demographic variability in treatment patterns. Employment status emerged as a significant predictor of treatment rates, with retired individuals exhibiting the highest rates, followed by unemployed individuals. Interestingly, treatment rates were highest among retired individuals, followed by unemployed individuals. This pattern could reflect a higher burden of comorbidities in these groups or greater availability to attend healthcare services, whereas working individuals may encounter time constraints that reduce healthcare utilization and treatment adherence. These findings are consistent with prior evidence suggesting that time availability and structural access barriers influence health service utilization and medication adherence in working-age populations [14]. On the other hand, this observation may be indicative of the heightened prevalence of cardiovascular risk factors in older populations and the potential influence of unemployment on health behaviors and access to healthcare resources. These findings align with previous research indicating that patients facing socioeconomic barriers to healthcare experience disparities, leading to worse outcomes and higher mortality rates from atherosclerotic cardiovascular disease [15]. Additionally, significant regional differences were observed in the use of lipid-lowering therapies across Spain’s Autonomous Communities. These findings are consistent with patterns previously described by Pedro-Botet et al., who examined dyslipidemia control and the use of lipid-lowering therapies in patients at high or very high cardiovascular risk across various regions in Spain [2]. The higher prescription rates observed in smaller municipalities may reflect the greater proportion of elderly residents in rural areas or the possibility of more personalized follow-up in lower-density healthcare settings [16,17]. Conversely, the lower prescription rates in regions with higher GDP per capita, such as Catalonia or the Basque Country, could be influenced by regional health policy strategies, healthcare delivery models, or stricter cost-control measures [18]. However, the database does not include information on clinical outcomes or healthcare efficiency, and such interpretations should be considered speculative [2]. These disparities underscore the necessity for targeted interventions to ensure equitable access to lipid-lowering therapies and address the underlying factors contributing to regional variability. The significant variation in the use of combination therapies across regions further emphasizes the need for standardized clinical guidelines and protocols, such as those provided by the ESC/EAS. These guidelines recommend combination lipid-lowering therapy—typically statins combined with ezetimibe or PCSK9 inhibitors—for patients at high or very high cardiovascular risk, in order to achieve more stringent LDL-C targets (<70 or <55 mg/dL, depending on risk stratification) [19]. This is consistent with findings from large European studies such as the DA VINCI and SANTORINI studies, which revealed that LDL-C goal attainment remains suboptimal among high- and very high-risk patients. The DA VINCI study showed that only 33% of patients reached the 2019 ESC/EAS guideline targets, with combination therapies used in just 10% of cases [20]. Similarly, the SANTORINI study reported that only 20% of patients achieved LDL-C goals, with combination treatment prescribed to just 24% of participants, highlighting persistent treatment gaps across Europe [21]. These findings have significant implications for clinical practice and healthcare policy. The growing use of lipid-lowering therapies, especially combination treatments, highlights the critical role of controlling lipids in reducing cardiovascular risk. Studies have shown that combining statins with other agents is both safe and effective in reducing LDL-C levels and reducing cardiovascular risk [22,23]. Clinicians should be encouraged to adhere to evidence-based guidelines and consider combination therapies when clinically appropriate [8,24]. Addressing the observed socioeconomic and regional disparities in lipid-lowering drug prescriptions requires a multifaceted approach. Socioeconomic factors, including poverty, education, and access to healthcare insurance, have been shown to contribute significantly to disparities in cardiovascular health outcomes [22,25]. It is imperative that policymakers prioritize enhancing access to healthcare for vulnerable populations through targeted outreach initiatives, enhanced coordination between primary and specialized care, and the implementation of standardized clinical protocols nationwide [26]. Geographical, knowledge, and regulatory barriers significantly impede access to lipid-lowering therapies, further exacerbating disparities in healthcare infrastructure and affordability [25].

While this study provides valuable insights into the use of lipid-lowering therapies for primary prevention in Spain, several limitations must be acknowledged. First, the reliance on the BDCAP database, although comprehensive, inherently excludes information on key aspects of dyslipidemia management, such as lifestyle interventions, dietary habits, physical activity, and smoking cessation, which are critical components of cardiovascular risk reduction. Previous research has highlighted that EHRs, while valuable for large-scale cardiovascular disease surveillance, often lack detailed data on these essential lifestyle factors, potentially limiting a full understanding of patient management strategies [27]. In addition, studies indicate that EHR systems may not consistently capture data on non-pharmacological interventions, contributing to gaps in assessing real-world cardiovascular risk management [28]. Moreover, the BDCAP database does not account for medication adherence or persistence, which are vital for understanding the real-world effectiveness of lipid-lowering therapies. Incomplete adherence data is a well-documented limitation of EHRs, as patient self-reported adherence is rarely recorded, and pharmacy dispensing data may not accurately reflect actual medication intake [29]. Additionally, the cross-sectional design of the study limits the ability to infer causality or examine long-term cardiovascular outcomes of the observed treatment patterns. Furthermore, given the administrative nature of the BDCAP database, the completeness and quality of the recorded data depend on the information systems of each Autonomous Community. The database does not provide access to the percentage of missing values for each variable, which may affect the interpretation of regional results and warrants cautious interpretation. The absence of clinical variables related to individual cardiovascular risk profiles limits the ability to fully interpret the prescribing differences observed by sex and age. These data are not available within the BDCAP statistical portal, and their absence is a limitation inherent to the database structure. On the other hand, although this study focuses exclusively on lipid-lowering prescriptions, the use of other pharmacological groups or the comprehensive cardiovascular risk profile of patients could provide additional insights into prescribing practices. Finally, regional variations in data quality and coding practices might introduce bias, potentially influencing the observed disparities in treatment rates and outcomes between Autonomous Communities. Another important limitation is that the BDCAP database does not provide information on the specific type or dosage of lipid-lowering agents prescribed. Consequently, we were unable to analyze the distribution of individual drug classes (e.g., statins by intensity, ezetimibe, PCSK9 inhibitors) or the detailed composition of combination therapies. This limits the granularity of the pharmacological analysis and should be considered when interpreting the results. Moreover, BDCAP data capture only prescribed medications, without confirmation of pharmacy dispensing or patient retrieval. As a result, discrepancies between prescribed and dispensed drugs—reported in other real-world settings—could not be assessed. This gap may partly account for the mismatch between high prescription rates and suboptimal LDL-C goal attainment. Given these limitations, future studies should aim to incorporate longitudinal data, integrate adherence metrics, and include detailed assessments of lifestyle modifications to provide a more comprehensive evaluation of lipid-lowering therapy effectiveness and cardiovascular risk reduction. Moreover, due to the aggregation of data under the general ATC C10A code, it is not possible to determine whether combination therapies consisted of dual regimens (e.g., statin plus ezetimibe) or more complex combinations involving bempedoic acid or PCSK9 inhibitors. This limits the specificity of our pharmacological analysis and should be considered when interpreting the results. Taken together, these limitations suggest the need for future studies to incorporate longitudinal designs, adherence data, and detailed lifestyle factors to fully understand real-world lipid-lowering treatment effectiveness.

Despite these limitations, the study has notable strengths. To our knowledge, this is the first study in Spain to comprehensively analyze the use of lipid-lowering therapies specifically for primary prevention using a robust, representative national database. The inclusion of demographic, socioeconomic, and geographic variables provides a nuanced understanding of treatment disparities, while the stratified analysis offers valuable insights into gender, age, and regional differences. Prior studies have highlighted that racial and ethnic minorities are less likely to be prescribed lipid-lowering therapies, emphasizing the importance of considering demographic factors in treatment analysis [30]. Additionally, disparities in statin prescription rates have been observed, with Black and Hispanic individuals being prescribed these therapies less frequent than White individuals, further underscoring the role of socioeconomic factors in access to treatment [31]. Geographic differences in the response and adherence to lipid-lowering therapies have also been documented, supporting the need for region-specific approaches to improve treatment strategies [32]. Furthermore, the temporal analysis of combination therapy trends highlights an important shift in clinical practice toward more intensive lipid management strategies. The increasing use of combination lipid-lowering therapies in recent years reflects a move toward more aggressive cardiovascular risk reduction, aligning with recommendations for stricter LDL-C targets in high-risk populations [33].

This study underscores the pivotal role of lipid-lowering therapies in primary prevention and highlights the need for targeted interventions to address socioeconomic and regional disparities. The notable increase in the use of combination therapies reflects a shift towards more intensive treatment strategies, achieving better lipid control in patients. However, the findings also reveal significant regional differences in treatment rates and the adoption of combination therapies, underscoring the need for more equitable healthcare delivery across Spain. By adhering to evidence-based guidelines, promoting the broader adoption of combination therapies where clinically appropriate, and implementing standardized clinical protocols, healthcare providers and policymakers can work towards a more effective and equitable approach to dyslipidemia and cardiovascular risk management nationwide.

## Figures and Tables

**Table 1:** Patients taking lipid-lowering drugs in 2023.

	Total Number	Per Thousand Attended
**Total**	7,373,297	167.96
**Gender**		
Women	3,789,865	166.63
Men	3,583,433	169.38
**Type of drug**		
Plain	6,241,581	149.48
Combined	1,131,716	24.2
**Type of prevention**		
Primary prevention	5,807,868	139.64
Secondary prevention	1,565,429	678.0

**Table 2:** Patients taking lipid lowering drugs in 2023 in primary prevention.

	Total Number	Per Thousand Attended	*p*
**Total**	5,807,868	139.64	
**Gender**			
Women	3,228,947	148.21	
Men	2,578,922	130.21	<0.01
**Size of the municipality**			
<10,000 inhab.	1,039,095	162.36	<0.001
10,001–50,000 inhab.	1,765,079	127.36	
50,001–100,000 inhab.	828,615	122.57	
100,001–500,000 inhab.	1,338,874	128.16	
>500,000 inhab.	836,207	119.68	
**Employment status**			
Employed	1,456,408	82.87	
Unemployed	325,622	121.92	
Inactive	647,168	51.64	
Retired	3,054,465	400.46	<0.001
Other	324,205	80.03	

**Table 3:** Type of lipid lowering drugs taken by year (2019–2023) in primary prevention.

		2019	2020	2021	2022	2023
**Plain drug**	Persons with drug	4,595,119	4,533,839	4,829,340	4,823,337	5,312,405
	Persons with drug per thousand assigned	106.35	104.68	110.83	114.96	119.50
**Combined**	Persons with drug	313,089	337,092	426,734	359,716	690,837
	Persons with drug per thousand assigned	7.25	7.78	9.79	10.42	15.54
**Total**	Persons with drug	4,908,208	4,870,931	5,256,074	5,183,053	6,003,242
	Persons with drug per thousand assigned	113.60	112.47	120.63	117.72	135.04

**Table 4:** People taking lipid lowering drugs by age and sex in primary prevention (2023).

	Men		Women		Total	
	Total Number	Per Thousand Attended	Total Number	Per Thousand Attended	Total Number	Per Thousand Attended
**30–34 years**	18,028	13.09	11,568	8.43	29,596	10.76
**35–39 years**	39,230	26.40	21,320	14.06	60,550	20.17
**40–44 years**	87,712	49.52	47,532	26.77	135,244	38.13
**45–49 years**	169,893	84.66	105,205	53.34	275,098	69.13
**50–54 years**	252,927	137.74	214,907	116.08	467,834	126.86
**55–59 years**	338,221	207.18	361,787	209.48	700,008	208.36
**60–64 years**	381,919	284.19	455,450	302.18	837,369	293.70
**65–69 years**	377,623	363.64	489,242	387.20	866,865	376.57
**70–74 years**	335,080	421.29	470,756	449.61	805,836	437.38
**75–79 years**	278,763	446.70	440,475	482.93	719,238	468,21
**80–84 years**	166,502	436.06	304,152	480.14	470,654	463.56
**85–89 years**	86,525	377.39	196,572	420.25	283,097	406.15
**90–94 years**	29,596	282.31	82,280	310.91	111,876	302.80
**≥95 years**	3637	146.72	14,505	167.63	18,142	162.97

**Table 5:** Lipid lowering drugs by region in 2023 in primary prevention.

	Population Attended	Persons with Drug	Percentage with Drug	Plain Drug	Combined Drug	Percentage with Combined Drugs over Total Taking Lipid Lowering Drugs
**Andalucía**	7,908,922	1,017,200	12.86%	884,749	132,451	14.97%
**Aragón**	1,281,053	204,618	15.97%	181,447	23,171	12.77%
**Asturias**	926,723	151,201	16.32%	130,718	20,483	15.67%
**Baleares**	1,145,766	124,159	10.84%	111,313	12,846	11.54%
**Canarias**	2,000,880	301,081	15.05%	248,577	52,504	21.12%
**Cantabria**	540,606	98,185	18.16%	85,736	12,449	14.52%
**Castilla y León**	2,171,741	389,103	17.92%	337,754	51,349	15.20%
**Castilla la Mancha**	1,936,658	338,916	17.50%	292,936	45,980	15.70%
**Cataluña**	7,290,688	645,619	8.86%	617,244	28,375	4.60%
**Comunidad Valenciana**	4,643,138	652,315	14.05%	582,034	70,281	12.08%
**Extremadura**	987,095	175,590	17.79%	144,436	31,154	21.57%
**Galicia**	2,456,338	475,208	19.35%	411,993	63,215	15.34%
**Madrid**	6,640,044	856,412	12.90%	781,479	74,933	9.59%
**Murcia**	1,476,759	223,204	15.11%	189,210	33,994	17.97%
**Navarra**	622,949	76,899	12.34%	69,570	7329	10.53%
**País Vasco**	2,120,550	200,078	9.44%	180,203	19,875	11.03%
**La Rioja**	305,207	37,613	12.32%	32,384	5229	16.15%
**Total**	**44,455,249**	**5,967,401**	**13.42%**	**5,281,782**	**685,619**	**12.98%**

## Data Availability

All data generated in this study are available from the corresponding author upon reasonable request. Additionally, some data can be accessed through the following website: https://www.sanidad.gob.es/estadEstudios/estadisticas/estadisticas/estMinisterio/SIAP/home.htm (accessed on 21 August 2025).
